# Uric Acid Correlates with Serum Levels of Mineral Bone Metabolism and Inflammation Biomarkers in Patients with Stage 3a–5 Chronic Kidney Disease

**DOI:** 10.3390/medicina60122081

**Published:** 2024-12-19

**Authors:** Francisco Mendoza Carrera, Gloria Elizabeth Vázquez Rivera, Caridad A. Leal Cortés, Lourdes del Carmen Rizo De la Torre, Renato Parra Michel, Rosalba Orozco Sandoval, Mariana Pérez Coria

**Affiliations:** 1 División de Medicina Molecular, Centro de Investigación Biomédica de Occidente (CIBO), Instituto Mexicano del Seguro Social (IMSS), Guadalajara 44340, Jalisco, Mexico; gloria.vazquez@alumno.udg.mx (G.E.V.R.); lourdes.rizo@imss.gob.mx (L.d.C.R.D.l.T.); dra.marianacoria@gmail.com (M.P.C.); 2 División de Investigación Quirúrgica, Centro de Investigación Biomédica de Occidente (CIBO), Instituto Mexicano del Seguro Social (IMSS), Guadalajara 44340, Jalisco, Mexico; lealc36@yahoo.com.mx; 3 Servicio de Nefrología, Hospital General Regional No. 46, Instituto Mexicano del Seguro Social (IMSS), Guadalajara 44910, Jalisco, Mexico; renocito2004@yahoo.com.mx; 4 Unidad de Medicina Familiar No. 3, Instituto Mexicano del Seguro Social (IMSS), Guadalajara 44340, Jalisco, Mexico; rosalba.orozco@imss.gob.mx

**Keywords:** uric acid, chronic kidney disease, mineral bone disorder, parathormone, FGF23, inflammation

## Abstract

*Background and Objectives*: Uric acid (UA) and the markers of mineral bone metabolism and inflammation are commonly altered in patients with chronic kidney disease (CKD) and are associated with the risk of cardiovascular complications and death. Studies point to a link between high serum UA and mineral bone homeostasis and inflammation, but controversy remains. The aim of this study was to evaluate the relationship between UA levels and mineral bone metabolism and inflammation biomarkers in a sample of Mexican patients with CKD 3a–5. *Materials and Methods*: This cross-sectional study included 146 Mexican patients with CKD 3a–5. In addition, 25 healthy subjects were included in the study with the aim of generating reference data for comparisons. Metabolic parameters including UA serum concentrations, mineral bone metabolism (parathormone (PTH), fibroblast growth factor 23 (FGF23), calcium, and phosphate), and inflammation (interleukin (IL)-1β, IL-6, and tumor necrosis factor-alpha (TNF-α)) biomarkers were measured in all of the samples and compared as a function of the estimated glomerular function rate (eGFR) or UA levels. *Results*: Intact PTH, FGF23, and cytokines were higher in advanced CKD stages. Patients with hyperuricemia had significantly higher values of FGF23 and TNF-α compared with those without hyperuricemia. The eGFR was found to be significantly and negatively correlated with all markers. Uric acid was significantly correlated with phosphate, iPTH, FGF23, and TNF-α, whereas iPTH was significantly correlated with FGF23, TNF-α, and FGF23. Finally, a multivariate analysis confirmed the relationship of eGFR with all the tested biomarkers, as well as other relationships of iPTH with UA and TNF-α and of FGF23 with UA and TNF-α. *Conclusions*: This study supports the relationship between uric acid and levels of mineral bone metabolism and inflammation biomarkers in patients with CKD at middle to advanced stages. In the follow-up of patients with CKD, monitoring and controlling UA levels through nutritional or pharmacological interventions could help in the prevention of alterations related to mineral bone metabolism.

## 1. Introduction

Chronic kidney disease (CKD) is defined as persistent alterations in kidney structure, function, or both, with implications for health and a duration of at least 3 months, regardless of the underlying cause [1,2]. Through the course of the decline in kidney function, a variety of alterations are faced by patients with CKD. Impairments in the excretion of molecules that are harmful to the body, on the one hand, and in the reabsorption of those that are beneficial, on the other, cause a series of systemic responses that subsequently have a negative impact on the health of the patients with CKD. In addition, the kidney is an organ with a key role in the homeostasis of bone and mineral metabolism, and once kidney function declines, the ability to regulate the excretion and absorption of molecules related to bone metabolism, such as calcium and phosphate, is also affected [3]. Mineral bone disorder (MBD) is a common condition in patients with CKD at middle to advanced stages; it is characterized by hyperphosphatemia, hypocalcemia, and elevated levels of parathormone (PTH) and is associated with bone disease, vascular calcifications, and risk of all-cause and cardiovascular death [4]. Consequently, the international Kidney Disease Improving Global Outcomes (KDIGO) guidelines (2017) for CKD-MBD suggest maintaining serum calcium, phosphate, and PTH within normal ranges to reduce the high mortality risk in these patients, although more research about these and other MBD biomarkers is encouraged [5]. One of these is the fibroblast growth factor 23 (FGF23), the levels of which negatively correlate with the glomerular function rate (GFR) in patients with CKD [6,7], and increased levels of this molecule have been associated with endocrine and metabolism dysregulation, as well as cardiovascular alterations [8,9]. FGF23 is considered a phosphatonine because of its contribution to protecting the organism against increased phosphate concentration by inhibiting its tubular absorption [10]. Together with PTH and vitamin D, FGF23 conforms to the PTH–vitamin D–FGF23 axis, which is key in maintaining phosphate levels in the organism for proper bone metabolism [11,12]. Additionally, recent experimental and clinical studies have shown that FGF23 can be involved in systemic inflammation, hypertension, iron metabolism, and cardiovascular disease, among other comorbidities associated with CKD [7,9,13,14,15].

A hallmark of CKD is a persistent and systemic inflammation state through all of the stages, which contributes to the development of various complications observed in CKD patients [16]. Moreover, a microinflammatory state has been suggested as an important risk factor for mortality in CKD [16,17]. Therefore, certain inflammatory biomarkers, such as interleukin (IL)-1, IL-18, IL-6, tumor necrosis factor-alpha (TNF-α), and C-reactive protein, among others, have been reported to be used as possible predictors and therapeutic targets for CKD [16]. In recent years, uric acid (UA) has gained attention because of its implication in several clinical conditions, including CKD. Uric acid is the end product of purine metabolism in mammals, and, because two-thirds of urate excretion is carried out by the kidney in humans, increased levels of serum UA or hyperuricemia (defined as serum UA levels of >6.0 mg/dL for females and >7.0 mg/dL for males) are commonly found in patients with CKD [18]. Hyperuricemia may lead to the deposition of urate crystals in the joints, kidneys, and other areas, resulting in joint and kidney damage [19]. In patients with hyperuricemia without gout, high levels of proinflammatory cytokines have been reported, suggesting close links between uric acid, inflammation, and renal damage [20]. Additionally, studies show that UA could be implicated in mineral bone metabolism [21,22] because it has been found to be associated with susceptibility to bone fractures by inhibiting the action of PTH and vitamin D during calcium uptake [21,23]. Patients receiving a recombinant PTH, teriparatide, show dose-dependent elevated UA levels [24]. Conversely, hyperuricemia is drastically reduced after parathyroidectomy [25], reinforcing the suggested link between PTH and UA balance. In this scenario, the mechanism by which hyperuricemia is related to MBD remains under discussion and a dual effect of the UA seems to be underway. Studies in patients with gout and kidney disease have found that increased UA levels reduce the serum 1,25(OH)2D3 by inhibiting 1α-hydroxylase activity, leading to decreased intestinal calcium absorption, which, in turn, inhibits the formation of osteoblasts and increase bone resorption [26]. On the other hand, a positive association between vitamin D status and UA has also been reported [27,28], suggesting a protective effect of increased urate levels in stabilizing bone mass [29,30]. Despite its apparent dual effect, authors have suggested that controlling UA levels could prevent common complications in patients with CKD [25], although clinical trials have shown controversial results [31].

The Mexican population has one of the highest rates of CKD worldwide [32], and some studies on mineral bone metabolism markers and hyperuricemia in healthy subjects and patients with CKD have been conducted [33,34], but the relationship with mineral bone metabolism has not been reported in this population. In this study, the association between UA levels and mineral bone metabolism markers, such as PTH and FGF23, as well as inflammation, was analyzed in a group of Mexican patients with CKD 3a–5.

## 2. Materials and Methods

### 2.1. Study Design and Subjects

A cross-sectional study was conducted, and it included 146 patients with a diagnosis of CKD 3a–5 without dialysis requirements who were recruited from the Nephrology Service of a second-level medical unit of the Mexican Institute of Social Security in Guadalajara, Mexico. Patient recruitment took place from June 2021 to May 2022, and the criteria for CKD diagnosis and classification were according to the KDIGO guidelines [2]. Patients with specific treatment for hyperuricemia or gout, lymphoproliferative cancer, systemic disease, a history of acute infection of any type, or an acute inflammatory condition two weeks prior to sample collection were excluded from this study. In addition, 25 apparently healthy subjects were included with the aim of having reference values of all analyzed biomarkers. These patients acknowledged themselves as healthy or denied having a previous diagnosis of chronic kidney disease, hypertension, or diabetes at the time of recruitment. Biochemical and anthropometric measurements were conducted for both the patients and healthy subjects. The estimated glomerular filtration rate (eGFR) was calculated with the CKD-EPI formula [35]. The presence of proteinuria or albuminuria was considered only when data for the urine albumin value greater than 30 mg/g creatinine was available from the clinical record or if the result of the general urinalysis was reported as positive for proteinuria. The body mass index was calculated by applying the following standard formula: body weight expressed in kilograms divided by the height in square meters (kg/m^2^). Diagnosis of diabetes was established according to the American Diabetes Association (ADA) [36]. Diagnosis of hypertension was established according to the American College of Cardiology/American Heart Association Task Force on Clinical Practice Guidelines [37], or if antihypertensive drugs were used at the time of recruitment. For the CKD patients, the cause of kidney disease and pharmacological management were taken from the hospital records. This study was approved by the Research Ethics Board of the Instituto Mexicano del Seguro Social (approval number: R-2021-1305-007, by 24 May 2021). All patients agreed to their enrollment in the study and signed a letter of informed consent.

### 2.2. Biochemical Parameters

Peripheral blood was obtained via venous puncture in the morning after a 12 h fast. After centrifugation of the samples at 4000 rpm for 20 min at 4 °C, the serum samples were separated. A portion of the serum was used for the measurement of common biochemical parameters, including glucose, creatinine, UA, total cholesterol, high-density cholesterol (HDL), triglycerides, calcium, inorganic phosphate, and total alkaline phosphatase. These were all measured by using standard dry chemistry with an automated clinical analyzer (Vitros 250 Integrated System, Ortho Clinical Diagnostics, Raritan, NJ, USA) according to the manufacturer’s instructions. The other portion of the sample was stored at −80 °C until further analysis. After thawing, all of the serum samples were used for the measurement of intact PTH, FGF23, interleukin (IL)-1β, IL-6, and TNF-α using a Bone Metabolism Multiplex Assay Kit (Merck-Millipore, Darmstadt, Germany), which was read in a Luminex 200 instrument (Austin, TX, USA).

### 2.3. Statistical Analysis

Categorical variables are presented as numbers and percentages, while numerical variables are shown as means ± standard deviation or medians and interquartile ranges (IQRs), respectively, according to the normality of the distribution after the Kolmogorov–Smirnov test was applied. Comparisons between groups were conducted by using the Chi-square for categorical variables, while Student’s *t*-test, the Mann–Whitney *U*-test, or the Kruskal–Wallis test was used for quantitative variables. Bivariate (with two-tailed Pearson bivariate analysis) and multiple linear regressions were conducted to analyze the data of our population and metabolites. Back-wise models were run to evaluate the relationship of UA concentrations with mineral bone metabolism and inflammation biomarkers. Only parameters with statistical significance in the bivariate correlation were included in the models, and only the variables that remained meaningful are reported in our results. Data analysis was performed using the SPSS statistical package for Windows, version 24.0, and *p* < 0.05 was considered significant.

## 3. Results

The clinical, anthropometric, and biochemical characteristics of the analyzed patients and healthy subjects are presented in Table 1. The CKD etiology was diabetic kidney disease (*n* = 134; 92%), followed by focal and segmentary glomerulosclerosis (*n* = 10; 7%), and two of undetermined etiology. Hypertension was observed in most of the patients (80%). Males were represented in higher numbers than females in this study. Compared with the healthy group, patients with CKD were older and had a higher prevalence of obesity. Uric acid levels and hyperuricemia were significantly higher in CKD patients than in the healthy group. Regarding mineral metabolism markers, there were higher levels of phosphate, tALKP, and FGF23 but lower levels of calcium in patients with CKD than in healthy subjects. Likewise, the levels of proinflammatory cytokines were higher in the patients.

**Figure 1:**
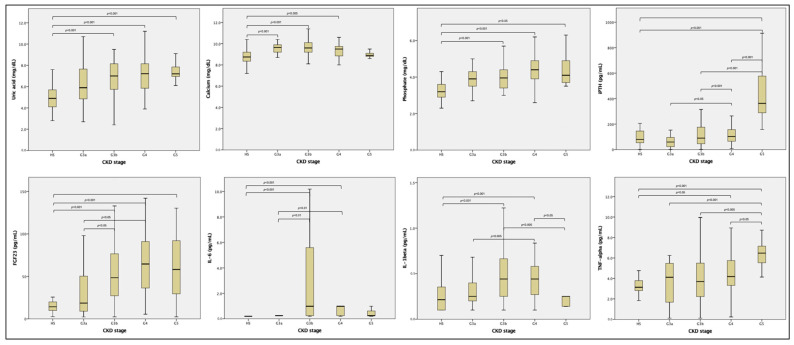
Serum concentrations of uric acid, mineral bone metabolism, and inflammation biomarkers according to the CKD stage. The *p-*values are obtained from ANOVA or Kruskal–Wallis tests, as appropriate. Abbreviations: HS: healthy subjects; iPTH, intact parathyroid hormone; FGF23, fibroblast growth factor 23; IL, interleukin; TNF, tumor necrosis factor.

Patients with CKD were grouped according to the presence of hyperuricemia, which was considered when SUA > 6.0 mg/dL if female or >7.0 mg/dL if male [18] and all of the parameters were compared accordingly (Table 2). Patients with hyperuricemia had lower eGFR and significantly higher levels of FGF23 and TNF-α than those without hyperuricemia. Comparisons of serum concentrations of UA, mineral bone metabolism, and inflammation markers according to the CKD stage are presented in Table 3 and Figure 1. Data for healthy subjects were included for reference. In general, all biomarkers were lower in the healthy subjects, as well as in patients with CKD in stage G3, with the exception of iPTH, which was similar in stages G3 to G4 (median < 120 pg/dL) but increased dramatically in stage G5 (median > 430 pg/dL). Uric acid was lower in healthy subjects and higher in patients at CKD stages G4 and G5. The calcium levels were relatively similar among all CKD stages and healthy subjects, with slight but significant differences between healthy subjects and stages G3-G4. The phosphate levels showed increased trends throughout the CKD stages, with significant differences only between healthy subjects and patients with CKD stages G3b-G5. A similar behavior was observed for FGF23 levels and TNF-α, although the greater differences were observed only at advanced stages (G4 and G5) for the proinflammatory cytokine. Regarding IL-1β and IL-6, some differences were observed, although these were rather marginal. Because it has been shown that some drugs commonly used in the treatment of CKD patients, such as diuretics and antihypertensives (angiotensin II receptor blockers, losartan) have a urate-lowering effect, we evaluated their association on UA levels and hyperuricemia, but no significant associations were found.

To evaluate the correlations between all of the markers of interest, including eGFR, a Pearson analysis was carried out, considering both CKD patients and healthy subjects as a whole (Table 4). As expected, eGFR was found to be significantly and negatively correlated with all markers, although in the cases of IL-1β and IL-6, the correlations were both rather weak and without statistical significance. The strongest correlations observed were of those of eGFR with UA (*r* = −0.439; *p* < 0.001), with phosphate (*r* = −0.446; *p* < 0.001), with FGF23 (*r* = −0.353; *p* < 0.001), and with TNF-α (*r* = −0.290; *p* < 0.001). Uric acid was significantly correlated with phosphate (*r* = 0.319; *p* < 0.001), with iPTH (*r* = 0.196; *p* = 0.010), and with FGF23 (*r* = 0.284; *p* < 0.001), and TNF-α (*r* = 0.169; *p* < 0.027); phosphate correlated with iPTH (*r* = 0.177; *p* = 0.030) and with FGF23 (*r* = 0.271; *p* = 0.001).

Finally, a multiple linear regression was performed to evaluate the relationships of only the variables with significant correlations in the previous bivariate analysis. For this, each biomarker was considered a dependent variable. Table 5 shows the final models listed in hierarchical order, with phosphate, iPTH, FGF23, and TNF-α as the only variables with statistical significance in the multivariate regression. The estimated GFR was the common variable that maintained its negative correlations with all of the tested biomarkers. In particular, eGFR was the only correlated variable for both phosphate [*B* = −0.020 (95% confidence interval (CI) −0.031, −0.008)] and UA [*B* = 0.013 (95%CI −0.018, −0.007)]. In addition, the correlation of iPTH with eGFR, UA, and TNF-α, the correlation of FGF23 with eGFR and UA, and the correlation of TNF-α with eGFR were also confirm.

## 4. Discussion

This study analyzed the relationships between UA levels and biomarkers of mineral bone metabolism and inflammation in patients with CKD in stages 3a–5 without dialysis requirements. The prevalence of hyperuricemia was rather high in CKD patients, reaching >50% of patients with this alteration, whereas this trait was found in less than 10% of a group of healthy subjects, as previously reported [19]. It is known that high UA is an independent predictor of CKD and could be implicated in the progression of CKD and complications related to other metabolic disorders, such as diabetes or hypertension [18,38], even in healthy subjects without comorbidities [19]. In the first analysis, significant correlations of UA levels with mineral bone metabolism biomarkers such as iPTH and FGF23 were observed. Investigations have shown that increased UA levels are associated with high levels of biomarkers of mineral metabolism, such as iPTH or phosphate in both healthy and CKD patients, independently of the CKD stage [34,39,40,41,42]. A recent large survey found that a significant relationship between UA and bone mineral density occurs starting in the early to intermediate stages of CKD and that this relationship is more significant in males, but mineral bone metabolism biomarkers were not measured [43]. This seems to agree with our observations, although our study included only intermediate-to advanced-stage patients; however, together with other authors, we could suggest that the relationship of UA–kidney function–mineral bone metabolism–inflammation could occur through all CKD stages [4], although more research is needed.

Hyperuricemia is a strong contributor to MBD; it is a very common condition in patients with CKD and is characterized by hyperphosphatemia, hypocalcemia, and elevated levels of PTH, which is associated with bone disease, vascular calcifications, and risk of all-cause and cardiovascular death [4,22,41]. To counteract this, international standard recommendations focus on maintaining serum calcium, phosphate, and PTH within normal ranges to reduce the mortality risk in these patients [5,44]. Since a patient with CKD is not a static entity, it is expected that the metabolic or biochemical parameters will vary over time due either to the normal course of the disease or to other factors that affect the patient’s clinical condition, such as comorbidities, the cause of renal failure, therapeutic scheme, and diet, among others. Our results suggest that the alteration of mineral bone biomarkers is ongoing in our sample of patients and although a diagnosis of MBD itself was not determined, the monitoring of the levels of these biomarkers could contribute to avoiding complications in the middle or long term. The mechanism linking UA with PTH is currently under discussion, but it is known that patients with primary parathyroidism show increased urate levels, and a rapid decline of urate levels has been seen after parathyroidectomy in experimental and clinical studies [25]. Additionally, the antiosteoporosis agent teriparatide, a recombinant PTH, has been found to promote hyperuricemia in a dose-dependent manner in post-menopausal women [24]. In this regard, it is known that the influence of UA on mineral metabolism is age- and sex-related [28,29,45]. Compared with men, women would be at an advantage at young ages due to a protective effect of estrogens on the UA transporters regulation by the kidney, but the risk of hyperuricemia and its related comorbidities equalize after menopause [46]. In our study, despite most of patients with CKD being male, there was no difference in age or sex when the presence of hyperuricemia was considered, perhaps due to advanced age in most of the patients (>65 years in both groups), which agree with previous observations [29,45]. Additionally, serum UA levels have also been associated with vitamin D insufficiency, another important factor in mineral bone metabolism in the middle-aged and elderly [26]. Unfortunately, vitamin D was not measured in this study, which could allow us a better assessment of the mineral bone status of our patients.

Another protagonist in mineral bone metabolism is FGF23, which plays an important role in protecting the organism against increased phosphate concentration by decreasing its tubular absorption [10]. FGF23 was indeed positively correlated with UA in the multivariate analysis, indicating an increase in this phosphatonine as UA increased. This result agrees with studies in which increasing levels of this protein correlate with kidney function decline [47,48]. The association of FGF23 and UA is not clearly understood, but one could suggest that it is likely secondary to the increase in phosphate or iPTH, as mentioned earlier, although only slight differences in these variables were found in our studied patients, which was perhaps due to the reduced sample size. Nonetheless, periodic measurements of FGF23 from the early stages of CKD could be useful in detecting individuals at high risk of major complications. In a longitudinal study in which levels of FGF23 were measured yearly, it was observed that individuals with rapidly rising FGF23 trajectories had a high risk of death compared with those that retained stable levels along the course of the study [39,49]. In contrast, experimental and clinical studies have demonstrated a relationship between inflammation markers such as TNF-α and IL-1β and FGF23 production [15,50], and a putative causal relationship between FGF23 levels and bone mineral density has been suggested [51]. Bone densitometry studies in the general population have linked increased UA with increased bone mineral density in a physiological state [22,30,52], although the precise relationship between these two parameters is not clear, since it is affected by the interplay of a range of variables, such as the patient’s age, gender, and diet [28,34,53]. Although information about bone status, such as bone mineral density, fracture history or other bone events, were not registered in this patient sample, our results agree with the association of UA levels with mineral bone metabolism and inflammation biomarkers in the context of CKD. Follow-up and interventional studies including larger sample size of both patient and healthy individuals will help to clarify the relationship of UA with mineral bone metabolism.

At least two explanations have been proposed for the observed link between hyperuricemia and inflammation. First, increased levels of UA could directly promote urate crystal formation and local inflammation response, which occurs in patients with gout [54], although studies in individuals with hyperuricemia who do not necessarily have gout have shown that cytokines such as IL-1β, IL-6, and TNF-α are increased as well [55]. Second, UA has been implicated in cellular damage by promoting oxidative stress and inflammation. Although UA is recognized as an antioxidant molecule, this beneficial property is exerted at the intracellular level. On the contrary, UA is a strong promoter of oxidative stress in the extracellular environment [21]. It has been proposed that oxidative stress and inflammatory cytokines induced by UA stimulate osteoclast bone resorption, which, along with UA’s regulation of vitamin D production, increases the risk of osteoporosis [21]. Alternatively, patients with SHPT show increased levels of inflammation mediators, such as TNF-α and IL-6 [3]. The fact that our patients were free of urate-lowering therapy at the time of recruitment allows us to suggest that the association of UA and the serum levels of the cytokines analyzed in this study could be due to something other than promotion by urate crystal formation (direct)—perhaps through oxidative-stress-induced inflammation—although oxidative stress was not evaluated. All of these data and the results from our study support the associations between UA, inflammation, and mineral bone metabolism. Studies on the precise cellular and physiological mechanisms are necessary to gain a better understanding of this phenomenon.

Because of the close relationship of UA and CKD, it has been suggested that controlling UA levels could prevent some of the major complications seen in patients with CKD, such as cardiovascular disease [25]. Several clinical trials have shown that pharmacologic urate-lowering therapy may slow the kidney function decline in patients with CKD and hyperuricemia and prevent cardiovascular risk, but its efficacy is still inconclusive [18,31]. On the other hand, drugs other than allopurinol and febuxostat have been shown with lowering UA levels action, such as the angiotensin II receptor blocker, losartan [18]. It is known that losartan has uricosuric activity by preventing the urate reabsorption through the urate transporter 1 (URAT1) blockade, but it has also been reported that its uricosuric action in hypertensive patients is influenced by variants in the URAT1 gene [56,57], some of these highly frequent in some populations [58]. In our study, patients receiving lowering-urate treatment, such as allopurinol or febuxostat, were excluded, but most patients (>50%) received losartan as antihypertensive therapy; however, no association with hyperuricemia nor UA levels were found. Beside the constraints due to the small sample size, we cannot rule out the influence of genetic factors in this population.

Our study has strengths and weaknesses. First, the patients were recruited from a single hospital unit, thus avoiding bias due to differences in clinical management or populational diversity given that all of them were of the same ethnicity (Mexican), although a genetic study was not carried out. Second, all of the analytes, including iPTH, FGF23, and cytokines, were measured at once and from the same sample, which allowed us to limit the increased inter-assay variations in comparison with if they had been measured separately. Given the cross-sectional nature of the study design, it is not possible to establish the directionality of the observed associations. Future follow-up and periodical measurements in this same patient group could provide greater consistency to our observations. Third, although only patients who were not consuming urate-lowering medication were included, there could be another drug, such as those used for glucose control or for osteoporosis treatment, that could have influenced the uric acid concentrations or inflammatory markers [59]. Besides the reduced sample size, an important limitation was that data for albuminuria/proteinuria, diet or other lifestyle habits, such as alcohol or tobacco consumption were not considered in our analyses, which could give more strength to our study. Notwithstanding these limitations, our study sets a precedent in the assessment of bone metabolism and inflammation markers in Mexican patients with CKD and shows that the monitoring of the levels of these biomarkers could contribute to avoiding complications in the medium and long term in these patients.

## 5. Conclusions

Our study supports previous reports showing a relationship between uric acid and levels of mineral bone metabolism and inflammation markers in patients with CKD in the middle to advanced stages. In the follow-up of patients with CKD, monitoring or controlling UA levels through nutritional or pharmacological interventions could help in the prevention of alterations related to mineral metabolism.

## Figures and Tables

**Table 1:** Clinical and biochemical characteristics of patients with chronic kidney disease (CKD) and healthy subjects.

Variable	CKD Patients *n* = 146	Healthy Subjects *n* = 25	*p*-Value
Female/male, count (%)	61/85 (42/58)	16/9 (64/36)	0.039
Age (years)	67 ± 18	52 ± 10	<0.001
BMI (kg/m^2^)	27.2 ± 5.9	26.8 ± 2.9	0.300
With obesity ^a^, count (%)	49 (33.5)	2 (8)	0.009
SBP (mmHg)	140 ± 42	125 ± 11	<0.001
DBP (mmHg)	80 ± 15	70 ± 7	<0.001
With hypertension, count (%)	117 (80.3)	0	-
With type 2 diabetes, count (%)	134 (91.8)	0	-
Fasting glucose (mg/dL)	119 ± 60	96 ± 12	<0.001
Uric acid (mg/dL)	6.9 ± 1.6	5.0 ± 1.2	<0.001
With hyperuricemia ^b^, count (%)	86 (56)	2 (8)	<0.001
Total cholesterol (mg/dL)	170 ± 72	186 ± 48	0.566
HDL-C (mg/dL)	41 ± 12	40 ± 6	0.736
Triglycerides (mg/dL)	178 ± 109	143 ± 65	0.003
Creatinine (mg/dL)	2.1 ± 1.0	0.9 ± 0.2	<0.001
eGFR (mL/min/1.73 m^2^)	31 ± 19	94 ± 19	<0.001
With proteinuria ^c^ count (%)	71 (60.7) ^d^	0	-
Calcium (mg/dL)	9.5 ± 0.9	8.8 ± 0.7	<0.001
Phosphate (mg/dL)	4.1 ± 1.0	3.2 ± 0.5	<0.001
tALKP (U/L)	94 ± 45	75 ± 25	0.027
Intact PTH (pg/mL)	94 ± 110	97 ± 60	0.451
FGF23 (pg/mL)	48 ± 57	16 ± 9	<0.001
IL-1β (pg/mL)	0.4 ± 0.3	0.2 ± 0.3	0.001
IL-6 (pg/mL)	1.0 ± 0.7	0.2 ± 0.1	0.001
TNF-α (pg/mL)	4.3 ± 2.4	3.1 ± 1.0	0.002

Abbreviations: CKD, chronic kidney disease; BMI, body mass index; SBP, systolic blood pressure; DBP, diastolic blood pressure; HDL-C, high-density cholesterol; tALKP, total alkaline phosphatase; eGFR, estimated glomerular filtration rate; PTH, parathyroid hormone; FGF23, fibroblast growth factor 23; IL, interleukin; TNF, tumor necrosis factor. Numerical variables are shown as medians ± IQRs or means ± standard deviations according to a normality distribution test. ^a^ Obesity was defined as BMI ≥ 30 kg/m^2^. ^b^ Hyperuricemia was considered when SUA > 6.0 mg/dL if female or >7.0 mg/dL if male [18]. ^c^ Proteinuria was considered when urine albumin > 30 mg/g creatinine or positive for protein in urinalysis (see Section 2). ^d^ Only data from 117 patients were available.

**Table 2:** Comparison of the data according to the presence of hyperuricemia.

Variable	Hyperuricemia ^a^ *n* = 81	Normouricemia *n* = 65	*p*-Value
Female/male count (%)	39/42 (48/52)	22/43 (34/66)	0.093
Age (years)	66 ± 17	70 ± 17	0.227
BMI (kg/m^2^)	27.3 ± 9.9	27.2 5.9	0.999
With obesity, count (%)	26 (32)	23 (35)	0.726
SBP (mmHg)	143 ± 25	142 ± 28	0.836
DBP (mmHg)	79 ± 13	79 ± 12	0.825
With hypertension, count (%)	69 (85)	49 (75)	0.270
Fasting glucose (mg/dL)	115 ± 60	124 ± 59	0.096
HbA1C (%)	6.9 ± 3.4	7.3 ± 2.8	0.653
Total cholesterol (mg/dL)	166 ± 72	174 ± 77	0.896
HDL-C (mg/dL)	40 ± 10	41 ± 13	0.665
Triglycerides (mg/dL)	178 ± 106	181 ± 123	0.834
Creatinine (mg/dL)	2.2 ± 0.9	1.9 ± 1.0	0.129
eGFR (mL/min/1.73 m^2^)	29 ± 17	33 ± 21	0.096
Calcium (mg/dL)	9.5 ± 0.7	9.4 ± 0.8	0.647
Phosphate (mg/dL)	4.3 ± 0.9	3.4 ± 0.9	0.090
tALKP (U/L)	100 ± 37	109 ± 76	0.622
Intact PTH (pg/mL)	131.2 ± 128.3	82.1 ± 75.0	0.096
FGF23 (pg/mL)	59.6 ± 50.3	40.4 ± 50.3	0.004
IL-1β (pg/mL)	0.4 ± 0.3	0.3 ± 0.4	0.183
IL-6 (pg/mL)	1.0 ± 0.8	0.7 ± 0.6	0.943
TNF-α (pg/mL)	4.7 ± 2.3	3.8 ± 2.3	0.022

Abbreviations: BMI, body mass index; SBP, systolic blood pressure; DBP, diastolic blood pressure; HbA1C, glycated hemoglobin; HDL-C, high-density cholesterol; eGFR, estimated glomerular filtration rate; tALKP, total alkaline phosphatase; PTH, parathyroid hormone; FGF23, fibroblast growth factor 23; IL, interleukin; TNF, tumor necrosis factor. Numerical variables are shown as medians ± IQRs or means ± standard deviations as appropriate, and categorical variables are shown as counts. The tests for categorical and numerical variables included the Chi-square, Mann–Whitney *U-*test*,* or Student’s *t-*test. ^a^ Hyperuricemia was considered when SUA > 6.0 mg/dL if female or >7.0 mg/dL if male [18].

**Table 3:** Serum levels of biomarkers according to the CKD stage.

Variable	HSα *n* = 25	G3a *n* = 30	G3b *n* = 49	G4 *n* = 57	G5 *n* = 10
Uric acid (mg/dL)	5.0 ± 1.2	6.1 ± 1.7	7.0 ± 1.6	7.2 ± 1.6	7.2 ± 1.2
Calcium (mg/dL)	8.8 ± 0.7	9.6 ± 0.9	9.6 ± 0.7	9.4 ± 0.7	8.9 ± 0.6
Phosphate (mg/dL)	3.2 ± 0.5	3.8 ± 0.6	4.1 ± 0.8	4.4 ± 0.9	4.2 ± 1.4
Intact PTH (pg/mL)	97 ± 60	68 ± 57	117 ± 94	113 ± 71	436 ± 222
FGF23 (pg/mL)	16 ± 9	34 ± 32	62 ± 49	75 ± 64	68 ± 59
IL-1β (pg/mL)	0.2 ± 0.3	0.3 ± 0.3	0.5 ± 0.4	0.5 ± 0.4	0.2 ± 0.1
IL-6 (pg/mL)	0.2 ± 0.1	0.3 ± 0.1	1.0 ± 7.6	1.0 ± 0.7	0.3 ± 0.1
TNF-α (pg/mL)	3.1 ± 1.0	3.5 ± 2.1	4.0 ± 2.5	4.6 ± 2.5	6.8 ± 2.2

Values are the means ± standard deviations or medians ± IQR. Kruskal–Wallis or ANOVA tests, accordingly. Significant comparisons are presented in Figure 1. Abbreviations: HS, healthy subjects; PTH, intact parathyroid hormone; FGF23, fibroblast growth factor 23; IL, interleukin; TNF, tumor necrosis factor.

**Table 4:** Correlations between the main analyzed variables.

	eGFR	Uric Acid	Calcium	Phosphate	tALKP	iPTH	FGF23	IL-1β	IL-6	TNF-α
eGFR	-	−0.439	−0.271	−0.446	−0.298	−0.269	−0.353	−0.092	−0.060	−0.290
Uric acid	<0.001	-	0.266	0.319	−0.142	0.199	0.284	0.129	0.099	0.169
Calcium	0.001	0.001	-	0.024	0.185	−0.158	0.138	−0.051	−0.010	0.034
Phosphate	<0.001	<0.001	0.775	-	0.119	0.177	0.271	0.036	0.104	0.130
tALKP	0.021	0.280	0.157	0.367	-	0.180	−0.085	−0.172	−0.112	0.258
iPTH	<0.001	0.010	0.054	0.030	0.168	-	0.205	−0.059	0.020	0.387
FGF23	<0.001	<0.001	0.092	0.001	0.518	0.007	-	0.219	0.143	0.190
IL-1β	0.129	0.093	0.536	0.659	0.189	0.444	0.004	-	0.070	0.079
IL-6	0.099	0.200	0.905	0.204	0.396	0.850	0.062	0.363	-	0.015
TNF-α	<0.001	0.027	0.678	0.113	0.046	<0.001	0.013	0.304	0.849	-

Data are presented as Pearson’s correlation coefficients (above the diagonal) and *p*-values (below the diagonal). Abbreviations: tALKP, total alkaline phosphatase; iPTH, intact parathyroid hormone; FGF23, fibroblast growth factor 23; IL, interleukin; TNF, tumor necrosis factor.

**Table 5:** Multivariate linear regression models for mineral bone metabolism biomarkers in the entire sample.

**Uric Acid ^a^**		
	**B (95% confidence interval)**	***p***
eGFR (mL/min/1.73 m^2^)	−0.020 (−0.031, −0.008)	0.001
**Phosphate ^b^**		
	**B (95% confidence interval)**	***p***
eGFR (mL/min/1.73 m^2^)	−0.013 (−0.018, −0.007)	<0.000
**iPTH ^c^**		
	**B (95% confidence interval)**	***p***
TNF-α (pg/mL)	16.212 (−7.563, 24.861)	<0.000
Calcium (mg/dL)	−37.417 (−61.335, −13.49)	0.002
eGFR (mL/min/1.73 m^2^)	−1.218 (−2.015, −0.420)	0.003
**FGF23 ^d^**		
	**B (95% confidence interval)**	***p***
eGFR (mL/min/1.73 m^2^)	−0.484 (−0.771, −0.196)	0.001
Uric acid (mg/dL)	4.711 (0.327, 9.094)	0.035
**TNF-** **α** **^e^**		
	**B (95% confidence interval)**	***p***
iPTH (pg/mL)	0.006 (0.003, 0.009)	<0.000
eGFR (mL/min/1.73 m^2^)	−0.018 (−0.030, −0.005)	0.006

Abbreviations: eGFR, estimated glomerular filtration rate; iPTH, intact parathyroid hormone; FGF23, fibroblast growth factor 23; TNF, tumor necrosis factor. Note: R^2^ of the models: ^a^ 0.279, ^b^ 0.216, ^c^ 0.204, ^d^ 0.158, ^e^ 0.187. VIFs were calculated to avoid collinearity, and the maximum value was 1.6 for FGF23 with UA. The remaining parameters for all tested models are available upon request.

## Data Availability

The datasets used and/or analyzed during the current study are available from the corresponding author upon reasonable request.
